# The effects of intracranial volume adjustment approaches on multiple regional MRI volumes in healthy aging and Alzheimer's disease

**DOI:** 10.3389/fnagi.2014.00264

**Published:** 2014-10-07

**Authors:** Olga Voevodskaya, Andrew Simmons, Richard Nordenskjöld, Joel Kullberg, Håkan Ahlström, Lars Lind, Lars-Olof Wahlund, Elna-Marie Larsson, Eric Westman, Alzheimer's Disease Neuroimaging Initiative

**Affiliations:** ^1^Department of Neurobiology, Care Sciences and Society, Karolinska InstitutetStockholm, Sweden; ^2^Department of Neuroimaging, Institute of Psychiatry, King's College LondonLondon, UK; ^3^NIHR Biomedical Research Centre for Mental Health and NIHR Biomedical Research Unit for DementiaLondon, UK; ^4^Department of Radiology, Uppsala UniversityUppsala, Sweden; ^5^Department of Medical Sciences, Uppsala UniversityUppsala, Sweden

**Keywords:** intracranial volume, normalization, Alzheimer's disease, neuroimaging, gender dimorphism, healthy aging

## Abstract

In neurodegeneration research, normalization of regional volumes by intracranial volume (ICV) is important to estimate the extent of disease-driven atrophy. There is little agreement as to whether raw volumes, volume-to-ICV fractions or regional volumes from which the ICV factor has been regressed out should be used for volumetric brain imaging studies. Using multiple regional cortical and subcortical volumetric measures generated by Freesurfer (51 in total), the main aim of this study was to elucidate the implications of these adjustment approaches. Magnetic resonance imaging (MRI) data were analyzed from two large cohorts, the population-based PIVUS cohort (*N* = 406, all subjects age 75) and the Alzheimer disease Neuroimaging Initiative (ADNI) cohort (*N* = 724). Further, we studied whether the chosen ICV normalization approach influenced the relationship between hippocampus and cognition in the three diagnostic groups of the ADNI cohort (Alzheimer's disease, mild cognitive impairment, and healthy individuals). The ability of raw vs. adjusted hippocampal volumes to predict diagnostic status was also assessed. In both cohorts raw volumes correlate positively with ICV, but do not scale directly proportionally with it. The correlation direction is reversed for all volume-to-ICV fractions, except the lateral and third ventricles. Most gray matter fractions are larger in females, while lateral ventricle fractions are greater in males. Residual correction effectively eliminated the correlation between the regional volumes and ICV and removed gender differences. The association between hippocampal volumes and cognition was not altered by ICV normalization. Comparing prediction of diagnostic status using the different approaches, small but significant differences were found. The choice of normalization approach should be carefully considered when designing a volumetric brain imaging study.

## Introduction

Volumetric measurements are widely used to study morphological changes in both normal aging and in neurodegenerative disorders. Understanding changes in regional brain volumes has the potential to aid prediction of onset and progression of many neurodegenerative disorders. However, volumetric analysis of brain structures is confounded by the difficulty of dealing with inter-individual variability in brain morphology and total head size.

In volumetric studies using magnetic resonance imaging (MRI), values of intracranial volume (ICV) are often used as proxy variables for premorbid brain volume. ICV differs substantially between males and females (Blatter et al., 1995; Scahill et al., 2003; Raz et al., 2004), males having a 10–12% larger ICV (Dekaban, 1978; Buckner et al., 2004). The effect of age on ICV seems to be minimal (Scahill et al., 2003; Buckner et al., 2004). In neurodegeneration research, normalization of regional brain volumes by ICV is often performed in order to better estimate the extent of atrophy that is caused by pathology and is not a consequence of intrinsic gender differences or other factors.

One approach to compensate for head size variability is to include the total ICV as a covariate in the regression model. Other approaches involve normalizing volumetric data by either: (1) expressing the volumes of interest (VOI) as a proportion of ICV or (2) using residuals of a least-square derived linear regression between raw volumes and ICV to calculate adjusted volumes. O'Brien et al. discussed the reasoning behind the above-mentioned correction methods, illustrating the consequences of each in a pediatric cohort of patients and controls (O'Brien et al., 2011). Strengths and weaknesses of the proportion and the residual methods are also carefully assessed by Sanfilipo et al. (2004). The authors evaluate the ability of these normalization methods to adjust for various types of error in ICV and brain parenchymal volume estimates in a data set of multiple sclerosis patients and controls. Another study (Barnes et al., 2010) investigated the association between head size and a number of cerebral structures in a cohort of control subjects, ultimately concluding the need for head size correction in volumetric studies, while questioning the suitability of the proportional approach.

The issue of appropriate head size adjustment is also acknowledged in the context of age-related changes in cortical and subcortical structures in the healthy brain (Raz et al., 2004; Barnes et al., 2010; Walhovd et al., 2011). For detailed accounts of neuroanatomical age-related volume effects in healthy individuals (see Coffey et al., 1998; Good et al., 2001; Raz and Rodrigue, 2006; Sowell et al., 2007; Greenberg et al., 2008; Walhovd et al., 2011; Goodro et al., 2012). In Good et al. (2001), Raz et al. (2004), Barnes et al. (2010), Walhovd et al. (2011), Goodro et al. (2012) the age spans of the participants stretched from young adulthood into old age, whereas Greenberg et al. (2008) focused on an elderly population (age 60–85), as did Coffey et al. (1998). Naturally, variability in age of subjects is a prerequisite for studying the effect of aging on different anatomical structures. However, when the focus lies on determining the impact of different head-size adjustment strategies on the estimates of brain compartments, age becomes a confounder.

There is little agreement as to whether raw volumes, volume-to-ICV fractions or regional volumes from which the ICV factor has been regressed out should be used for volumetric brain imaging studies. This has previously only been investigated in relatively small cohorts using a limited set of predefined regions. Therefore, the main aim of the present study is to explore the relationship between multiple regional brain volumes and total ICV. In particular we are interested in describing how this relationship is influenced by various ICV normalization approaches. Further, the role of gender, age, cognition, and degree of pathology are investigated in connection to the normalization approaches. This is investigated in a total of 51 cortical and subcortical volumetric measures, using the Freesurfer pipeline, in a large sample (1130 subjects) from two different cohorts covering the spectrum from healthy elderly individuals (CTL), mild cognitive impairment (MCI) to Alzheimer disease (AD).

## Materials and methods

### Subjects and MRI

Two cohorts of subjects are studied in this paper.

The first cohort was collected as part of the Prospective Investigation of Vasculature in Uppsala Seniors (PIVUS) study (Lind et al., 2006). The subset of the PIVUS study with available MRI comprises of 406 cognitively normal elderly subjects residing in the community of Uppsala, Sweden. All subjects were scanned at the age of 75. Demographic details of the PIVUS cohort are presented in Table 1.

**Table 1:** **Demographics of the single-center epidemiological PIVUS cohort (left)**.

	**PIVUS cohort**			**PIVUS matched subset**	
	**Female**	**Male**	**All participants**	**Female**	**Male**
Number of participants	193	213	406	21	21
Intracranial volume (mm^3^)	1440320 (121240)	1638419 (140237)	1544249 (140237)	1570321^*b*^ (10971)	1570355^*b*^ (8816)
MMSE	28.8 (1.3)^*a*^	28.5 (1.5)^*a*^	28.7 (1.4)	29.0(1.0)	29.0(1.7)
Education					
<9 years	58%	58%	48%	43%	
9–12 years	18%	20%	19%	19%	19%
>12 years	24%	22%	23%	33%	38%
Age	75			75	

Demographics of the subset of the PIVUS cohort matched by ICV in addition to the inherent age-matching (right).

Data are presented as mean (standard deviation) where applicable.

MMSE, mini-mental state examination.

a Females had significantly higher MMSE than males at p < 0.05 (Mann-Whitney U-test: U = 22270).

b In the matched subsample comparing ICV_male_ and ICV_female_ yielded p = 0.96, Mann-Whitney U = 213.

MR images for the PIVUS study were acquired using a 1.5 Tesla clinical MRI scanner (Philips Healthcare, Best, The Netherlands). The MR protocol included a sagittal T1-weighted 3D gradient echo sequence (echo time: 4.0 ms, repetition time: 8.6 ms, resolution: 0.94 × 0.94 × 1.2 mm^3^).

The second cohort forms part of the ADNI dataset, which was downloaded from the ADNI database (http://adni.loni.usc.edu/, PI Michael M Weiner) (Jack et al., 2008). This cohort contains data from 223 CTL, 325 MCI, and 176 AD subjects. Details of the ADNI cohort are presented in Table 2.

**Table 2:** **Demographics of the multi-center ADNI cohort, divided into subsets according to the participants' diagnosis**.

	**CTL**			**MCI**			**AD**		
	**Female**	**Male**	**All**	**Female**	**Male**	**All**	**Female**	**Male**	**All**
Number of participants	107	116	223	124	201	325	86	90	176
Intracranial volume (mm^3^)	1444626 (130830)	1617014 (134231)	1534299 (157978)	1439337 (139318)	1643969 (123745)	1565894 (166451)	1423013 (116706)	1637473 (157880)	1532681 (175643)
MMSE	29.2(1.0)	29.0(1.0)	29.1(1.0)	26.8(1.8)	27.2(1.8)	27.1(1.8)	23.2(2.0)	23.4(2.0)	23.3(2.0)
ADAS-cog (Word recall)	2.5(1.1)^a^	3.2(1.1)^a^	2.9(1.1)	4.4(1.5)	4.6(1.3)	4.5(1.4)	6.0(1.5)	6.3(1.3)	6.1(1.4)
Education									
<9 years	2%	2%	2%	3%	1%	2%	6%	3%	5%
9–12 years	15%	4%	9%	19%	15%	17%	32%	22%	27%
>12 years	83%	94%	89%	78%	84%	81%	62%	74%	68%
Age	76.1 (4.8)	75.8(5.3)	75.9(5.1)	73.4(7.2)^b^	75.2(7.0)^b^	74.5(7.1)	74.7(7.6)	75.5 (7.1)	75.1(7.3)

The education level was stratified in a way that would make it comparable to the PIVUS cohort.

Data are presented as mean (standard deviation) where applicable.

CTL, cognitive normal; MCI, mild cognitive impairment; AD, Alzheimer's disease.

a There was a significant difference in ADAS-cog scores between males and females in the CTL group. (Welch two-sampled t-test, t = 4.8, df = 219.3, p < 0.0001).

b There was a significant difference in age between males and females in the MCI group. (Welch two-sampled t-test, t = 2.3, df = 251.2, p = 0.03).

MR images for ADNI were collected from a variety of 1.5 Tesla MR systems, using protocols optimized for each MR scanner. The standardized protocol included a high resolution sagittal 3D T1-weighted MPRAGE volume (echo time: 4.0 ms, repetition time: 9 ms, resolution: 1.1 × 1.1 × 1.2 mm^3^) acquired using a custom pulse sequence specifically designed for the ADNI study to ensure compatibility across scanners (Jack et al., 2008). Full brain and skull coverage was required for the MRI datasets and detailed quality control was carried out on all MR images according to previously published quality control criteria (Simmons et al., 2009, 2011).

### ICV estimation and regional subcortical volume segmentation

The FreeSurfer (version 5.1.0) pipeline was used to generate cortical and subcortical volumetric measures (Dale et al., 1999; Fischl et al., 1999). Estimated Total Intracranial Volume (eTIV) generated by FreeSurfer was used as an estimate for ICV in this study. The eTIV measure from FreeSurfer is in good agreement with ICV reference segmentation acquired from proton density weighted images) (Nordenskjöld et al., 2013) and has previously been used in several studies for normalization (Westman et al., 2011, 2012, 2013). The pipeline generated 68 cortical volumes (34 from each hemisphere) and 46 subcortical volumes. Volumes of white matter hypointensities, optic chiasm, right and left vessel, and left and right choroid plexus were excluded from further analysis. Cortical and subcortical volumetric measures from the right and left side were averaged (Walhovd et al., 2011; Westman et al., 2013). In total 34 regional cortical volumes and 17 subcortical volumes were used for final analysis in the study. Image processing steps were visually inspected (skull-stripping errors and gray/white matter boundary) to ensure they had been carried out correctly.

## Statistical procedures

### Head size normalization approaches

In this section we briefly present the background of the most common head size normalization methods, focusing on the practical implications for population-based and research cohorts. Theoretical analyses of head size adjustment strategies can also be found in Arndt et al. (1991), Sanfilipo et al. (2004).

The proportion approach calculates the ratio between the VOI and total ICV, producing a unitless value between 0 and 1. Further analyses, such as group comparisons are carried out using this outcome measure.

The second normalization method, which we refer to as the residual approach uses a linear regression between the VOI and ICV to predict the ICV-adjusted volumes. Adjusted volumes are obtained as follows:

(1) $${\text{Volume}}_{\text{adjusted }i}={\text{Volume}}_{\text{raw }i}-\beta ({\text{ICV}}_{\text{raw }i}-{\text{ICV}}_{\text{mean}})$$

where β is the slope of the regression line between ICV and the volume of interest (Jack et al., 1998).

In other words, ICV-adjusted volumes are generated from linear regression residuals:

(2) $$\begin{array}{l} {\text{Volume}}_{\text{adjusted }i}\;=\;{\text{Volume}}_{\text{raw }i}\;-\;\beta \left({\text{ICV}}_{\text{raw}}\;i\;-\;{\text{ICV}}_{\text{mean}}\right)\\ \;=\underset{\text{VOI-E}\left(\text{VOI}\right)={\text{residuals}}_{i}}{\underset{︸}{{\text{Volume}}_{\text{raw }i}-\beta \cdot {\text{ICV}}_{i}}}+\underset{\text{mean volume}}{\underset{︸}{\beta \cdot {\text{ICV}}_{\text{mean}}}} \end{array}$$

(3) $${\text{Volume}}_{\text{adjusted }i}\;=\;{\text{residual}}_{i}\;+\;{\text{Volume}}_{\text{mean}}$$

These outcome volumes are calculated from residuals of a least-square derived linear regression between raw volumes and ICV and are therefore statistically uncorrelated with ICV. In the case of comparing two groups, such as patients and controls, β from the controls is used to correct both groups. The assumption behind this is that the regression slope β represents the “normal” relationship between the VOI and ICV and that this relationship is not necessarily sustained in the case of pathology. Thus, when performing residual correction in the ADNI cohort, ICV estimates for the CTL subset were used to obtain adjusted regional volumes in the MCI and the AD subsets.

### Additional statistical procedures

In order to assess the correlation that exists between ICV and each regional volume Pearson correlation coefficients were computed between all subcortical and cortical volumes and ICV, using raw, proportional and residually-adjusted volumes. Volumetric gender comparisons were performed in the PIVUS cohort, using independent samples *t*-tests adjusted for multiple comparisons (Bonferroni correction). As a subsidiary analysis we tested whether differences in regional brain volumes between females and males are explained by males having larger total ICV, by identifying a subsample of the PIVUS cohort consisting of 21 males and 21 females that in addition to having the same age were closely matched by total ICV (Table 1). The relative standard deviation of the ICV within each group was less that 1%. In this approach we eliminate the need to adjust for inter-subject brain size variability, by creating groups where this variability has been minimized.

Due to the non-normality of the data within the matched cohort, a Mann–Whitney *U*-test was performed for group comparison. Assuming that the subjects in the matched subset are representative of a larger population of males and females with very similar ICVs, we used a bootstrapping procedure to calculate a sampling distribution for the *t*-statistic for each regional structure.

Statistical analysis in the ADNI cohort was conducted analogously with that in the PIVUS cohort. However, since age varies in the ADNI cohort, partial correlation between volume of interest and ICV was performed controlling for age in cases where the correlation between age and VOI was significant.

Volumetric gender comparisons were only performed in the CTL subset of the ADNI cohort for the purpose of comparing these to the PIVUS findings.

A linear regression model was used to determine how well raw hippocampal volumes, proportionally, and residually corrected hippocampal volumes predict the scores of the first item of the ADAS-cog (Rosen et al., 1984), the Word Recall Task. Further, we ran an ANOVA to compare hippocampal volumes across diagnostic groups in the three different normalization settings, with Tukey's HSD test as the *post-hoc* procedure.

We created CTL vs. MCI, CTL vs. AD, and MCI vs. AD models, using raw, proportional and residual hippocampus volumes as predictors. The classification performance of each model was assessed by comparing the resulting areas under the receiver operating curves (AUC).

All statistical analyses were performed using R (R Foundation for Statistical Computing, Vienna Austria, www.r-project.org).

## Results

### The PIVUS cohort

**Figure 1:**
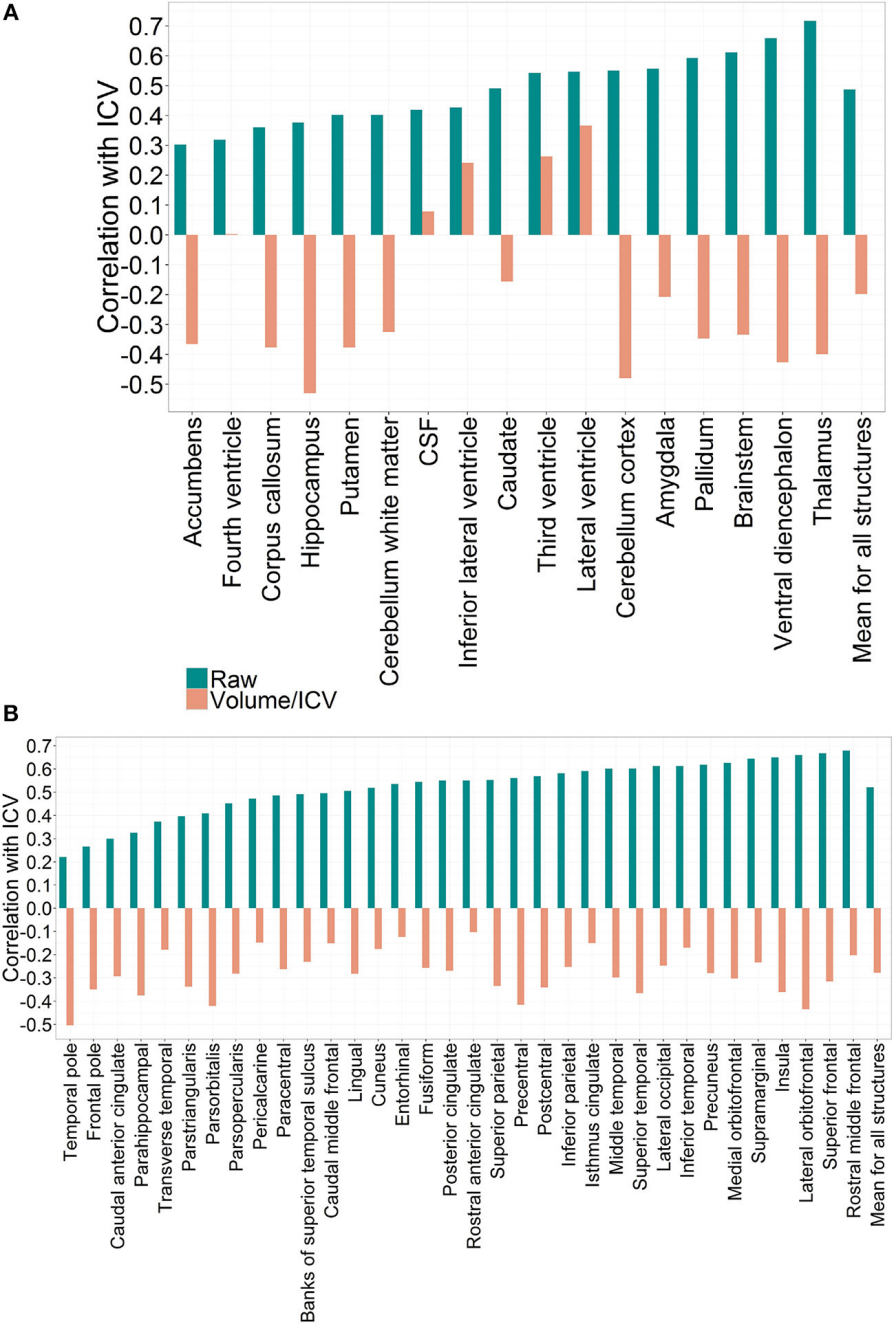
**(A)** Correlation patterns for individual regional subcortical volumes in the PIVUS cohort using different normalization methods. Mean correlation coefficient with ICV:R_raw_ = 0.49 (0.12), R_proportion_ = −0.20 (0.28), R_residual_ = 0. **(B)** Correlation patterns created by individual regional cortical volumes in the PIVUS cohort under different normalization settings. Mean correlation coefficient with ICV:R_raw_ = 0.52 (0.12), R_proportion_ = −0.28 (0.10), R_residual_ = 0.

Raw values of subcortical and cortical volume measurements are presented in Tables 3, 4. We found significant positive correlation between the ICV and all raw VOI. However, this correlation pattern was altered by the chosen normalization approach (Figure 1).

**Table 3:** **Subcortical volumes of the PIVUS cohort, presented separately for men and women with Bonferroni-adjusted results of gender comparisons**.

**Regional subcortical volumes (mm^3^)**	**Females ***N = 193*****		**Males ***N = 213*****		***t*-statistic**	***p*-value Bonferroni adjusted**
	**Mean**	**Standard deviation**	**Mean**	**Standard deviation**		
Lateral ventricle	16108	7352	22240	10083	7.0	<0.0001
Inferior lateral ventricle	691	400	1032	522	7.4	<0.0001
Cerebellum WM	13060	1846	13603	2055	2.8	0.09^*^
Cerebellum cortex	45579	4262	49411	5116	8.2	<0.0001
Thalamus	5711	562	6216	579	8.9	<0.0001
Caudate	3210	485	3506	612	5.4	<0.0001
Putamen	4275	592	4587	590	5.3	<0.0001
Pallidum	1418	165	1545	166	7.7	<0.0001
Third ventricle	1628	511	2113	671	8.2	<0.0001
Fourth ventricle	1647	585	1922	633	4.5	<0.001
Brainstem	19169	2051	20931	2401	8.0	<0.0001
Hippocampus	3405	379	3515	384	2.9	0.07^*^
Amygdala	1241	167	1401	181	9.2	<0.0001
CSF	1348	416	1609	463	6.0	<0.0001
Accumbens	439	69	471	70	4.7	<0.0001
Ventral DC	3415	315	3738	366	9.5	<0.0001
Corpus callosum	2638	363	2779	411	3.7	<0.01

* The unadjusted p-values are p = 0.004 for the cerebellum white matter and p = 0.005 for the hippocampus.

**Table 4:** **Cortical volumes of the PIVUS cohort, presented separately for men and women with Bonferroni-adjusted results of gender comparisons**.

**Regional cortical volumes (mm^3^)**	**Females *N = 193***		**Males *N = 213***		***t*-statistic**	***p*-value Bonferroni adjusted**
	**Mean**	**Standard deviation**	**Mean**	**Standard deviation**		
Banks of superior temporal sulcus	2034	291	2202	331	5.5	<0.0001
Caudal anterior cingulate	1792	298	1890	352	3.1	0.08
Caudal middle frontal gyrus	5167	870	5536	922	4.1	<0.01
Cuneus cortex	2386	334	2674	397	7.9	<0.0001
Entorhinal cortex	1757	268	2016	306	9.1	<0.0001
Fusiform gyrus	8077	1090	8842	1116	7.0	<0.0001
Inferior parietal cortex	11619	1522	12587	1591	6.3	<0.0001
Inferior temporal gyrus	9039	1177	10024	1343	7.9	<0.0001
Isthmus of cingulate cortex	2123	299	2388	319	8.7	<0.0001
Lateral occipital cortex	9638	1201	10551	1288	7.4	<0.0001
Lateral orbitofrontal cortex	6265	636	6711	628	7.1	<0.0001
Lingual gyrus	5169	705	5633	738	6.5	<0.0001
Medial orbitofrontal	4300	513	4667	504	7.3	<0.0001
Middle temporal gyrus	9508	1130	10275	1236	6.5	<0.0001
Parahippocampal gyrus	1794	258	1906	288	4.1	<0.01
Paracentral sulcus	2944	405	3160	478	4.9	<0.0001
Parsopercularis	3581	523	3757	543	3.3	<0.05
Parsorbitalis	2011	248	2123	264	4.4	<0.001
Parstriangularis	3059	414	3265	476	4.7	<0.001
Pericalcarine cortex	1691	293	1890	298	6.8	<0.0001
Postcentral gyrus	7895	927	8425	1018	5.5	<0.0001
Posterior cingulate	2710	351	2949	385	6.5	<0.0001
Precentral	10859	1156	11563	1302	5.8	<0.0001
Precuneus	7859	964	8643	946	8.3	<0.0001
Rostral anterior cingulate	2108	356	2348	334	7.0	<0.0001
Rostral middle frontal	12684	1532	13992	1622	8.4	<0.0001
Superior frontal gyrus	17886	1934	19609	2087	8.6	<0.0001
Superior parietal gyrus	11161	1366	11991	1427	6.0	<0.0001
Superior temporal	9630	1085	10290	1129	6.0	<0.0001
Supramarginal gyrus	8746	1093	9616	1094	8.0	<0.0001
Frontal pole	819	144	861	136	3.0	0.11
Temporal pole	2267	312	2348	302	2.6	0.30
Transverse temporal cortex	854	168	888	169	2.0	1
Insula	6219	648	6842	686	9.4	<0.0001

When expressed as proportions of total ICV all brain gray matter structures displayed consistent negative correlations with ICV. Third ventricle and lateral ventricles mantained their positive correlation with ICV even after division, whereas for the fourth ventricle and CSF any significant correlation was eliminated. The fact that applying the proportional normalization approach to regional gray matter volumes leads to over-correction suggests that these structures are not directly proportional to ICV.

Applying the residual method to correct for variability in ICV eliminated the correlation between ICV and volumes completely. This result is expected and arises from the fact that residual-adjusted volumes are calculated from residuals of a least-square derived linear regression between raw volumes and ICV, which are statistically uncorrelated with ICV (Arndt et al., 1991).

We found that all raw subcortical volumes were significantly greater in males than in females at *p* < 0.05 (Table 3). However, when adjusted for multiple comparisons using the Bonferroni correction, gender differences in cerebellum white matter and hippocampus were no longer significant. For cortical volumes, all regions except caudal anterior cingulate, frontal pole, temporal pole, and transverse temporal cortex were significantly greater in men than women (Table 4).

When expressed as fractions of total ICV, the majority of subcortical structures appeared to be significantly greater in women at *p* < 0.05 (Table 5). No significant differences between genders were found for caudate, fourth ventricle, amygdala, and CSF. Lateral and third ventricles remained larger for men even after ICV adjustment (Table 5). For cortical volumetric measures, the majority of the ICV-divided values also appeared to be greater for females (Table 6). Using the residual approach to eliminate all correlation between the ICV and cerebral structures led to the removal of any significant volume differences in all cortical and subcortical regions between females and males (Tables 5, 6).

**Table 5:** **Gender disparities in subcortical regional volumes in the PIVUS cohort and how they are affected by the different ICV normalization methods used**.

**Regional subcortical volumes (mm^3^)**	**Raw**			**Volume/ICV**			**Residual**		
	**Males > Females**		**Females > Males**	**Males > Females**		**Females > Males**	**Males > Females**		**Females > Males**
Lateral ventricle	✓			✓				–	
Inferior lateral ventricle	✓			✓				–	
Cerebellum white matter		–				✓		–	
Cerebellum cortex	✓					✓		–	
Thalamus	✓					✓		–	
Caudate	✓				–			–	
Putamen	✓					✓		–	
Pallidum	✓					✓		–	
Third ventricle	✓			✓				–	
Fourth ventricle	✓				–			–	
Brainstem	✓					✓		–	
Hippocampus		–				✓		–	
Amygdala	✓				–			–	
CSF	✓				–			–	
Accumbens	✓					✓		–	
Ventral DC	✓					✓		–	
Corpus callosum	✓					✓		–	

Results are based on Bonferroni-adjusted p-values.

**Table 6:** **Gender disparities in cortical regional volumes in the PIVUS cohort and how they are affected by the different ICV normalization methods used**.

**Regional cortical volumes (mm^3^)**	**Raw**			**Volume/ICV**			**Residual**		
	**Males > Females**		**Females > Males**	**Males > Females**		**Females > Males**	**Males > Females**		**Females > Males**
Banks of superior temporal sulcus	✓					✓		–	
Caudal anterior cingulate		–				✓		–	
Caudal middle frontal gyrus	✓					✓		–	
Cuneus cortex	✓				–			–	
Entorhinal cortex	✓				–			–	
Fusiform gyrus	✓				–			–	
Inferior parietal cortex	✓					✓		–	
Inferior temporal gyrus	✓				–			–	
Isthmus of cingulate cortex	✓				–			–	
Lateral occipital cortex	✓					✓		–	
Lateral orbitofrontal cortex	✓					✓		–	
Lingual gyrus	✓					✓		–	
Medial orbitofrontal gyrus	✓					✓		–	
Middle temporal gyrus	✓					✓		–	
Parahippocampal gyrus	✓					✓		–	
Paracentral sulcus	✓					✓		–	
Parsopercularis	✓					✓		–	
Parsorbitalis	✓					✓		–	
Parstriangularis	✓					✓		–	
Pericalcarine cortex	✓				–			–	
Postcentral gyrus	✓					✓		–	
Posterior cingulate	✓					✓		–	
Precentral	✓					✓		–	
Precuneus	✓				–			–	
Rostral anterior cingulate	✓				–			–	
Rostral middle frontal	✓				–			–	
Superior frontal gyrus	✓					✓		–	
Superior parietal gyrus	✓					✓		–	
Superior temporal	✓					✓		–	
Supramarginal gyrus	✓				–			–	
Frontal pole		–				✓		–	
Temporal pole		–				✓		–	
Transverse temporal cortex		–				✓		–	
Insula	✓				–			–	

Results are based on Bonferroni-adjusted p-values.

Comparing regional structures in the subset of 75 year olds matched by ICV, only significant difference between sexes remained in the volume of the third ventricle, where on average, males had a volume of 2090 mm^3^ (451 mm^3^) and females 1592 mm^3^ (366 mm^3^) (*p* = 0.019 after Bonferroni adjustment for multiple comparisons). None of the remaining cortical or subcortical volumes differed between men and women in this sample. Bootstrapping results were coherent with the above-mentioned findings. All simulation *p*-values were greater than 0.05, except in the case of the third ventricle, where *p* = 0.017 after Bonferroni correction.

### The ADNI cohort

**Figure 2:**
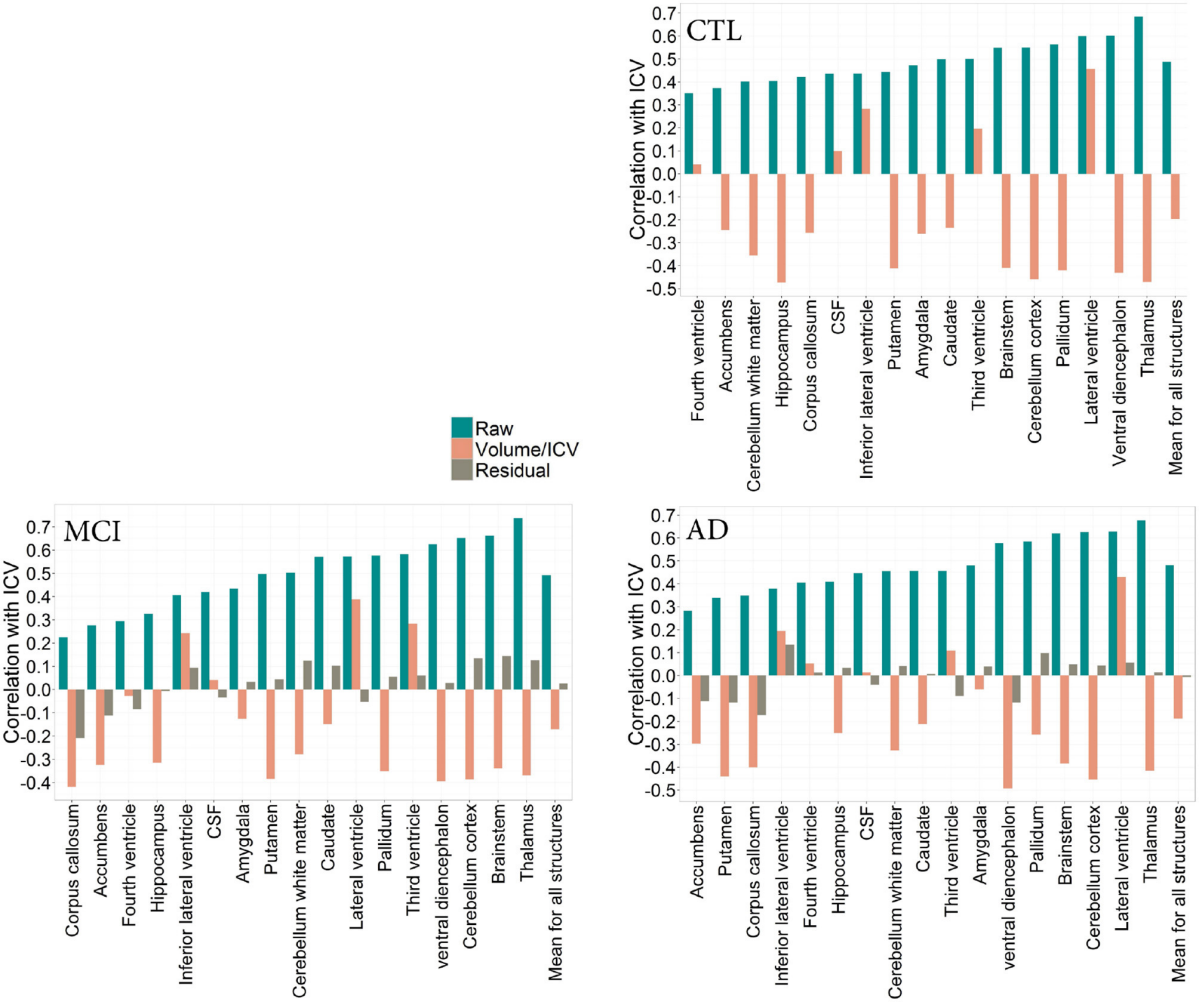
**CTL: Correlation patterns created for individual regional subcortical volumes in the CTL subset of the ADNI cohort using different normalization methods**. Mean correlation coefficients: R_raw_(CTL) = 0.49(0.10), R_proportion_(CTL) = −0.19(0.30) **MCI**: Correlation patterns created for individual regional subcortical volumes in the MCI subset of the ADNI cohort using different normalization methods. Mean correlation coefficients: R_raw_(MCI) = 0.49 (0.15), R_proportion_(MCI) = −0.17(0.26), R_residual_(MCI) = 0.02(0.10). **AD**: Correlation patterns created for individual regional subcortical volumes in the AD subset of the ADNI cohort using different normalization methods. Mean correlation coefficients: R_raw_(AD) = 0.49 (0.15), R_proportion_(AD) = −0.19 (0.27), R_residual_(AD) = 0.007 (0.09).

**Figure 3:**
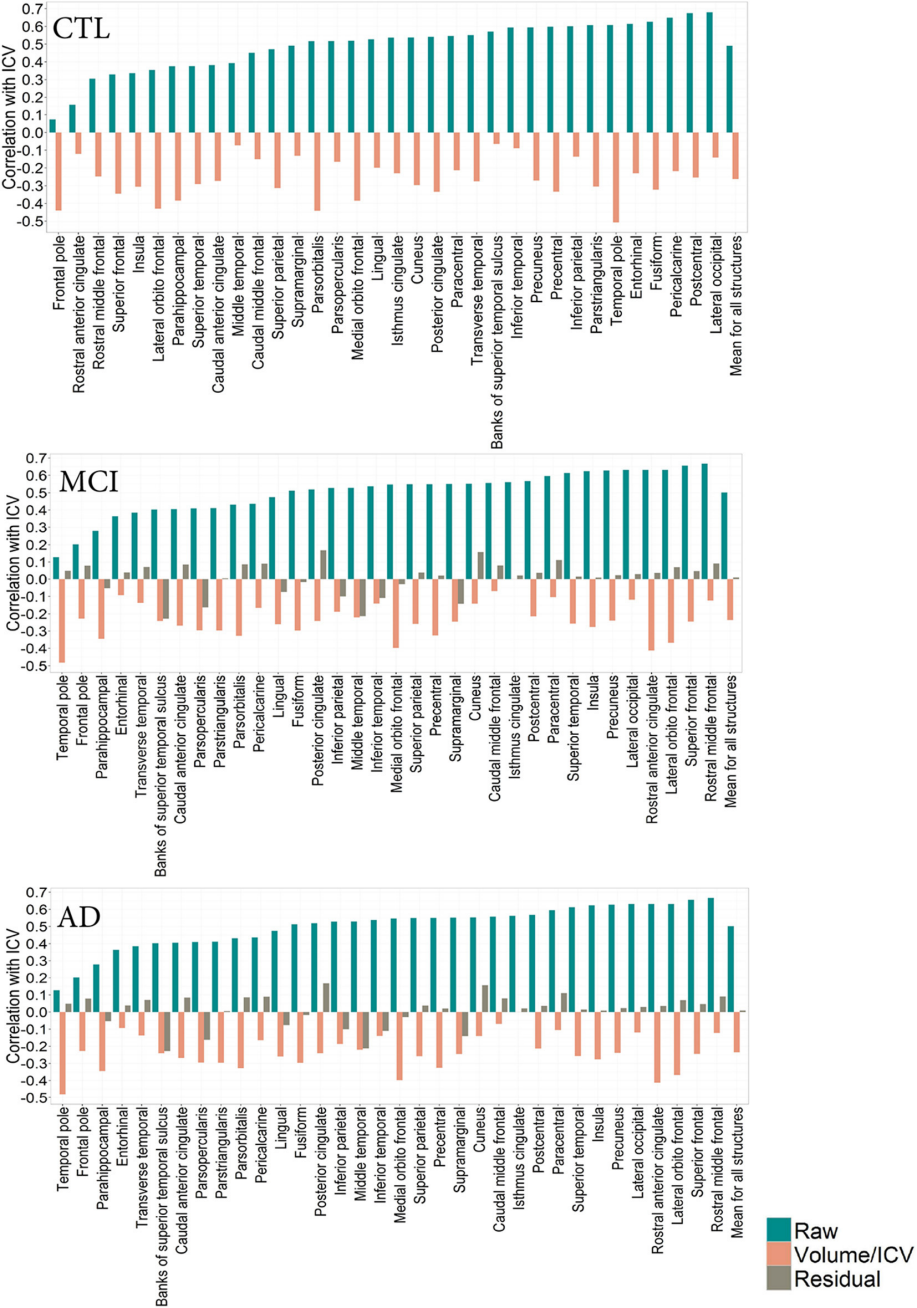
**CTL: Correlation patterns created for individual regional subcortical volumes in the CTL subset of the ADNI cohort using different normalization methods**. Mean correlation coefficients: R_raw_(CTL) = 0.49 (0.10), R_proportion_(CTL) = −0.19 (0.30). **MCI**: Correlation patterns created for individual regional subcortical volumes in the MCI subset of the ADNI cohort using different normalization methods. Mean correlation coefficients: R_raw_(MCI) = 0.49 (0.15)R_proportion_(MCI) = −0.17 (0.26), R_residual_(MCI) = 0.02(0.10). **AD**: Correlation patterns created for individual regional subcortical volumes in the AD subset of the ADNI cohort using different normalization methods. Mean correlation coefficients:R_raw_(AD) = 0.49 (0.15), R_proportion_(AD) = −0.19 (0.27) = 0.49 (0.15), R_residual_(AD) = 0.007(0.09).

To determine whether pathology has an effect on the relationship between total ICV and VOI across diagnostic groups, we describe this relationship in the CTL, MCI, and AD groups (Figures 2, 3). Since age is variable in the ADNI cohort, all correlation coefficients presented below are corrected for age, where the association between age and the volume of interest was significant. No association between age and ICV was found.

The correlation pattern within the ADNI cohort was qualitatively and quantitatively similar to that of the PIVUS cohort. Among subcortical regions, thalamus consistently had the highest positive correlation with ICV, irrespective of the diagnosis (r_CTL_ = 0.69, r_MCI_ = 0.74, r_AD_ = 0.68), the corresponding value for PIVUS is r_PIVUS_ = 0.72. Proportional normalization produced similar results as in the PIVUS dataset. VOI-to-ICV fractions for lateral ventricles sustained their positive correlation with ICV, whereas gray matter structures were overcorrected. Residual normalization applied to the AD and MCI subjects yielded an average correlation coefficient *r* close to zero, but did not eliminate the correlation with ICV completely. This is a consequence of using the β from the regression fit of the CTL data to normalize the MCI and AD data. The structures that maintained a significant correlation with ICV after residual correction were cerebellum white matter, cerebellum cortex, corpus callosum, thalamus, brainstem, and accumbens in the MCI subset and only the corpus callosum in the AD subset (Figure 2). For the correlation pattern in cortical structures see Figure 3.

Comparing male and female regional volumes in the CTL cohort we found that all raw subcortical volumes except caudate, CSF and corpus callosum were greater for males than females. Expressed as volume-to-ICV fractions, females had greater volumes of cerebellum white matter, thalamus, caudate, putamen, and pallidum. Males had greater proportional lateral ventricle volumes. The rest of the subcortical structures showed no significant differences between genders. Regressing out the ICV factor from the volumetric measures removed all structural differences between men and women. Gender comparisons for subcortical and cortical structures under different head size adjustments are presented in Tables 7, 8.

**Table 7:** **Gender disparities in subcortical regional volumes in the CTL subset of the ADNI cohort and how they are affected by the different ICV normalization methods used**.

**Regional subcortical volumes (mm^3^)**	**Raw**			**Volume/ICV**			**Residual**		
	**Males > Females**		**Females > Males**	**Males > Females**		**Females > Males**	**Males > Females**		**Females > Males**
Lateral ventricle	✓			✓				–	
Inferior lateral ventricle	✓			✓				–	
Cerebellum white matter	✓					✓		–	
Cerebellum cortex	✓				–			–	
Thalamus	✓					✓		–	
Caudate		–				✓		–	
Putamen	✓					✓		–	
Pallidum	✓					✓		–	
Third ventricle	✓				–			–	
Fourth ventricle	✓				–			–	
Brainstem	✓				–			–	
Hippocampus	✓				–			–	
Amygdala	✓				–			–	
CSF		–			–			–	
Accumbens	✓				–			–	
Ventral DC	✓				–			–	
Corpus callosum		–			–			–	

Results are based on Bonferroni-adjusted p-values.

**Table 8:** **Gender disparities in cortical regional volumes in the CTL subset of the ADNI cohort and how they are affected by the different ICV normalization methods used**.

**Regional cortical volumes (mm^3^)**	**Raw**			**Volume/ICV**			**Residual**		
	**Males > Females**		**Females > Males**	**Males > Females**		**Females > Males**	**Males > Females**		**Females > Males**
Banks of superior temporal sulcus	✓				–			–	
Caudal anterior cingulate		–			–			–	
Caudal middle frontal gyrus	✓				–			–	
Cuneus cortex	✓				–			–	
Entorhinal cortex	✓				–			–	
Fusiform gyrus	✓				–			–	
Inferior parietal cortex	✓				–			–	
Inferior temporal gyrus	✓				–			–	
Isthmus of cingulate cortex	✓				–			–	
Lateral occipital cortex	✓				–			–	
Lateral orbitofrontal cortex	✓				–			–	
Lingual gyrus	✓				–			–	
Medial orbitofrontal gyrus	✓				–			–	
Middle temporal gyrus	✓				–			–	
Parahippocampal gyrus	✓				–			–	
Paracentral sulcus	✓				–			–	
Parsopercularis	✓				–			–	
Parsorbitalis	✓					✓		–	
Parstriangularis	✓				–			–	
Pericalcarine cortex	✓				–			–	
Postcentral gyrus	✓				–			–	
Posterior cingulate	✓				–			–	
Precentral	✓				–			–	
Precuneus	✓				–			–	
Rostral anterior cingulate	✓				–			–	
Rostral middle frontal	✓				–			–	
Superior frontal gyrus	✓				–			–	
Superior parietal gyrus	✓					✓		–	
Superior temporal	✓				–			–	
Supramarginal gyrus	✓				–			–	
Frontal pole		–				✓		–	
Temporal pole		–				✓		–	
Transverse temporal cortex		–			–			–	
Insula	✓				–			–	

Results are based on Bonferroni-adjusted p-values.

We created three ADAS-cog~hippocampus linear regression models in order to establish which type of normalization approach is capable of explaining more of the variation within the ADAS-cog data. The three models had similar *R*^2^-values:

*R*^2^_raw_ = 0.154, *R*^2^_proportional_ = 0.211, *R*^2^_residually corrected_ = 0.196, although hippocampal volumes expressed proportionally to the ICV could explain slightly more of the variance in the ADAS-cog scores. A clear improvement to the raw data model was brought about by including the ICV×hippocampus interaction term in the model, increasing the *R*^2^ of the model to 0.212.

The three diagnostic groups in the ADNI cohort differed significantly in hippocampal volumes irrespective of the normalization method applied to the data. The relationship between ICV and the hippocampus was not significantly different across groups: r_CTL_ = 0.40, r_MCI_ = 0.33, r_AD_ = 0.41 (for comparison: r_PIVUS_ = 0.38). Linear regression models predicting hippocampal volumes, using total ICV and age as input were equally powerful for all groups: *R*^2^_CTL_ = 0.27, *R*^2^_MCI_ = 0.22, *R*^2^_AD_ = 0.25 with regression parameters β_CTL_ = 1.057e-03 β_MCI_ = 1.016e-03, β_AD_ = 1.118e-03. We investigated whether raw, ICV-divided or residually adjusted hippocampus data yielded the greatest AUC when used for classification in CTL vs. MCI, CTL vs. AD, and MCI vs. AD models (Table 9). We found that in distinguishing between CTL and MCI, ICV-divided hippocampus data produced an AUC that was significantly greater than the AUC for both raw and residual data. For the CTL vs. AD model, residual data performed significantly better than raw data. For discriminating between MCI and AD, using residual data yielded a significantly greater AUC than that for ICV-divided data.

**Table 9:** **Classification performance of the CTL vs. MCI, CTL vs. AD and MCI vs. AD models for the different normalizations of hippocampal data**.

	**Raw hippocampus volumes**	**Hippocampus/ICV**	**Residually corrected hippocampus volumes**
AUC: CTL vs. MCI^a^	0.73 [0.69–0.77]	0.76 [0.72–0.80]	0.75 [0.70–0.79]
AUC: CTL vs. AD^b^	0.88 [0.84–0.91]	0.89 [0.85–0.92]	0.90 [0.87–0.93]
AUC: MCI vs. AD^c^	0.69 [0.64–0.74]	0.66 [0.62–0.71]	0.70 [0.65–0.75]

Data are presented as mean [95% confidence interval]. CTL, cognitive normal; MCI, mild cognitive impairment; AD, Alzheimer's disease.

a In the CTL vs. MCI contrast, the area under the receiver operating curve (AUC) obtained from using hippocampus/ICV volumes was significantly greater comparing to the AUC obtained from both raw (p = 0.048) and residual (p = 0.037) hippocampus volumes.

b In the CTL vs. AD contrast, the AUC obtained from using residually corrected hippocampal volumes was significantly greater comparing to the AUC obtained from raw hippocampal volumes (p = 0.001).

c In the MCI vs. AD contrast, AUC obtained from using residually corrected hippocampal volumes was significantly greater comparing to the AUC obtained from hippocampus/ICV volumes (p < 0.001).

## Discussion

Our study addresses the association of multiple cortical and subcortical volumetric measures with total ICV. We describe the relationship between head size and its constituent regional volumes and examined how this relationship changes depending on the chosen ICV normalization method.

In the first part of this study we focused on the PIVUS cohort, containing cognitively normal participants all aged 75 at the time of the scan. Working with a large sample not confounded by age or pathology provided an excellent opportunity to study region-to-ICV dependence inherent to a healthy brain. Volumetric studies work under the rationale that larger brains comprise larger regional structures. Looking at raw volumes, we found that all cortical and subcortical structures showed positive correlations with ICV. In our sample, dealing with proportions instead of raw values reversed the direction of correlation with ICV for all cortical and subcortical structures, suggesting that the volumes of cerebral structures are not directly proportional to the total ICV. The fact that gray matter structures do not scale proportionately with total ICV volume has also been shown previously in Barnes et al. (2010). The main strength of this method is its use in calculating the VOI-to-ICV fraction for individual cases. A limitation of proportional volumes is that they encompass two sources of error, one originating from the numerator and one from the denominator. Further, it has been shown that an increase in correlation between the region of interest and the ICV leads to loss of reliability of the VOI-to-ICV fraction. A detailed examination of the proportional measures can be found in Arndt et al. (1991).

From gender comparisons performed in the population-based same aged cohort we found that all raw volumes of cerebral structures except the cerebellum white matter and the hippocampus were significantly larger in males than in females. Although there is an agreement across many studies that men possess larger cerebra than women (Gur et al., 1991; Blatter et al., 1995; Coffey et al., 1998; Raz et al., 2004), findings regarding the topography of gender-dimorphisms vary. Our findings that males had greater volumes in most structures, but not the hippocampus conform to the results in Barnes et al. (2010). Conversely, Greenberg et al. report a significantly larger hippocampal volumes in males than females, however showing that generally men did not have larger raw volumes despite having larger cerebra (Greenberg et al., 2008).

Expressed as region-to-ICV fractions we found that third and lateral ventricles constitute a significantly larger proportion of the male than the female brain. These findings could arise from: (1) men having congenitally smaller brain tissue-to-ventricle ratio than women, or (2) the fact that age-related volumetric decline has progressed further in men than women by the age of 75. The latter would entail an inclination toward a higher rate of volumetric decline in men and is supported by several studies (Gur et al., 1991; Blatter et al., 1995; Coffey et al., 1998; Good et al., 2001; Raz et al., 2004). In a volumetric analysis of 194 healthy males and females between ages 16 and 65, Blatter et al. found that ventricular expansion occurs at a faster rate and begins at an earlier age in men (Blatter et al., 1995). Both larger raw and ICV-divided ventricular volumes in men have also been found in Barnes et al. (2010). Another study focusing more directly on the aging brain revealed no difference in the rates of atrophy between men and women above the age of 65 (Walhovd et al., 2011). However, it is difficult to compare our results with findings from the above-mentioned studies. Particularly since the latter generally deal with smaller samples and examine fewer volumetric measures, and most importantly involve participants of variable ages. The fact that our findings are based on data from cognitively normal 75 year olds allows us to generalize for the population of this age. Although our dataset cannot provide concrete information about gender discrepancies in the rates of age-related volumetric decline, it allows us to infer that for this population the processes sustained by the male brain up until the age of 75 are distinct to those sustained by the female brain. It is important to state that further discrepancies among findings across studies arise from the difference in structural image quality, ICV adjustment method, and the applied segmentation techniques.

Working with residually corrected data effectively removed all correlations between regional volumes and ICV as well as the differences in cerebral substructures between men and women. Elimination of the gender effect after regressing out the total ICV has been demonstrated previously in Scahill et al. (2003), however not for such large numbers of regional structures. The rationale behind regressing out the ICV factor from the data can be questioned conceptually. By removing all variance associated with ICV from regional brain volumes we imply that absolute brain size is unimportant. This is contradictory to the brain reserve theory, which suggests that larger brain size can have protective effect in pathology. It has been shown in a sample of 270 AD patients that the impact of atrophy on cognition decreases with increasing head circumference (Perneczky et al., 2010). For an evaluation of the above-mentioned study and further discussion on the brain reserve concept (see Whitwell, 2010). In a more recent study using the ADNI cohort, it was found that greater ICV has some protective role in early AD (Guo et al., 2013). Whether we choose to accept or reject the brain reserve hypothesis, it is important to consider that removing all variance associated with head-size from VOI may also remove volume differences linked to protective or compensatory mechanisms.

Another way to compensate for brain size variability between subjects is to minimize this variability within the raw data. We apply this tactic to study whether larger absolute regional volumes in males are a consequence of the inherently larger total ICV of the male brain. In a selected sample of 21 males and 21 females matched closely by total ICV, the only significant difference between genders was the third ventricle, which was significantly larger for men. This could be an after-effect of one of the findings in Blatter et al. (1995), namely that the third ventricle shows a 60% higher correlation with age in men than in women. Naturally, matching subjects by age and ICV volume is an artificial approach, which favors females with above average ICV and males with below average ICV. Nevertheless, it is the only method that allows head-size variation to be removed.

A weakness of this study of the PIVUS cohort is the inability to investigate whether the observed gender disparities are a consequence of an earlier onset of age-related volumetric decline in men, a steeper rate of this decline, or whether they may be inborn. The strengths of this study are largely due to the unique properties of the PIVUS cohort in terms of both size and equal age of the subjects, as well as the uniformity and robustness of the MRI measurements. We therefore anticipate our findings to be generalizable to healthy men and women of this age.

In the second part of the study we investigated the relationship between ICV and cerebral substructures in the ADNI cohort to determine whether our findings from normal older subjects hold in the context of variable age and pathology. We found that the correlation pattern of the raw VOI and ICV was similar for the PIVUS dataset and the three diagnostic groups of the ADNI cohort. Based on our observations it seems that Alzheimer pathology does not influence the underlying relationship between ICV and brain volumes in a prominent way. Studying the gender discrepancies within the CTL group, a general agreement with the PIVUS findings were observed. The pattern that seems to hold across cohorts for both cortical and subcortical structures involves the majority of the raw volumes being greater for males, the majority of the proportional volumes being greater for females and no gender differences in regional volumes where the ICV has been regressed out. Several studies have reported that division by ICV significantly reduces the gender differences in global brain size measures (Kruggel, 2006; Smith et al., 2007). However, in the present study, where we analyze a large number of regional volumes we find that division does not reduce gender differences, but rather reverses their roles. Adjusting the values using the residual method eliminates all volumetric discrepancies between genders.

In the context of Alzheimer's disease neurodegeneration a special place is given to possible changes in hippocampal volume. Not surprisingly, the three diagnostic groups differ significantly in their hippocampal volume. However, the degree of association between the total ICV and the hippocampus is consistent across the groups. As a result of these inherent relationships being similar, proportional and residual ICV adjustments have the same effect on hippocampal data. Further we compared the ability of raw, divided, and residual hippocampus volumes to predict diagnosis status in CTL vs. MCI, CTL vs. AD, and MCI vs. AD models. Our results varied depending on the model: differences in classification performance were small, but significant and raw hippocampal data was never the most accurate predictor of diagnostic status.

In conclusion, the decision as to whether to use raw or ICV-adjusted volumetric data will affect the interpretation of any statistical analysis carried out in conjunction with a morphometric study. This seems to be especially true where gender comparisons are concerned. If there is no interest in the role of gender in a certain analysis, the residual method can be used, since it removes any volumetric gender-dimorphism. However, one should remember that this result is an underestimation, since true differences do exist. Division by ICV also has its advantages, mostly in terms of interpretability, since working with fractions allows conclusions to be drawn in a setting where there is a hypothesis about the proportion of ICV occupied by a certain region. Thus, in the case of ventricular volumes the information about their size in relation to the total ICV is more valuable than their volume in milliliters. The main advantage of working with raw volumes is their use in cross-study comparisons and the potential in creating normative volumetric values for different age spans. The choice of normalization approach should be carefully considered when designing a volumetric brain imaging study.

### Conflict of interest statement

The authors declare that the research was conducted in the absence of any commercial or financial relationships that could be construed as a potential conflict of interest.
